# Effects of *Porphyromonas gingivalis* lipopolysaccharide on osteoblast-osteoclast bidirectional EphB4-EphrinB2 signaling

**DOI:** 10.3892/etm.2013.1357

**Published:** 2013-10-23

**Authors:** YI ZHANG, XI-CHAO WANG, XING-FU BAO, MIN HU, WEI-XIAN YU

**Affiliations:** 1 Department of Orthodontics, School of Stomatology, Jilin University, Changchun, Jilin 130021, P.R. China; 2 Laboratory of Mechanism of Tooth Development and Bone Remodeling and Regeneration, School of Stomatology, Jilin University, Changchun, Jilin 130021, P.R. China

**Keywords:** *Porphyromonas gingivalis* lipopolysaccharide, EphB4, EphrinB2, bidirectional signaling

## Abstract

In bone remodeling, the Eph family is involved in regulating the process of osteoclast and osteoblast coordination in order to maintain bone homeostasis. In this study, the effects of *Porphyromonas gingivalis* lipopolysaccharide (Pg-LPS) on the osteoblast-osteoclast bidirectional EphB4-EphrinB2 signaling were investigated. An osteoblast-osteoclast co-culture system was achieved successfully. Hence, direct contact and communication between osteoblasts and osteoclasts was permitted. Regarding the protein expression and gene expression of EphB4 and EphrinB2, it was shown that Pg-LPS increased the expression of EphB4 while inhibiting the expression of EphrinB2. Therefore, the results indicate that, when treated with Pg-LPS, the EphB4 receptor on osteoblasts and the EphrinB2 ligand on osteoclasts may generate bidirectional anti-osteoclastogenic and pro-osteoblastogenic signaling into respective cells and potentially facilitate the transition from bone resorption to bone formation. This study may contribute to the control of osteoblast differentiation and bone formation at remodeling, and possibly also modeling, sites.

## Introduction

Bone remodeling is a coupling process of bone resorption and bone formation (1). Resorption by osteoclasts and formation by osteoblasts, which leads to the occurrence of a coupling mechanism, is a complex and life-long process (2). This remodeling process has been described as a ‘bone remodeling cycle’ consisting of activation, resorption, reversal and formation phases (3). It is crucial for the normal function of bone, including bone growth, bone repair and the replacement of obsolete bone. Therefore, the molecular mechanism of coupling has long been a focus of research in this area.

However, prior to the discovery of the effects of bidirectional Eph-ephrin signaling in bone homeostasis, no proper coupling mechanism was reported that was able to explain this process. Since its discovery 25 years ago, the Eph family of receptor tyrosine kinases, comprised of A- and B-subfamilies, has been found to be involved in a growing number of physiological and pathological processes in various cell types and organs (4,5). Notably, it has been confirmed that bidirectional Eph-ephrin signaling participates in many biological processes, including angiogenesis, bone and organizational development and axon guidance (6–10).

In bone remodeling, osteoclast and osteoblast coordination is the key to maintaining bone homeostasis. Ephrin is involved in regulating this process (11). It has been demonstrated that reverse signaling through EphrinB2 into osteoclast precursors suppresses osteoclast differentiation, while forward signaling through EphB4 into osteoblasts enhances osteogenic differentiation and the overexpression of EphB4 in osteoblasts increases bone mass in transgenic mice (12). This finding revealed the potential role of the Eph/ephrin receptor family of ligands in the bone. It has been suggested that EphrinB2 may act in a paracrine or autocrine manner on the osteoblast to stimulate osteoblast maturation and/or bone formation (13).

Chronic periodontitis, a major cause of anodontia in adults, is one of the most common oral diseases (14). *Porphyromonas gingivalis* (Pg) is recognized as the main pathogen in chronic periodontitis (15). Lipopolysaccharide (LPS) from Pg is a component of Gram-negative bacterial cell walls. *Porphyromonas gingivalis* lipopolysaccharide (Pg-LPS), with high toxicity and antigenicity to periodontal tissue, may lead to the loss of periodontal attachment and alveolar bone absorption (16,17). LPS has also been shown to be able to induce the formation of osteoclasts with bone resorbing activity in RAW 264.7 cells (18).

In the present study, the effects of Pg-LPS on osteoblast-osteoclast bidirectional EphB4-EphrinB2 signaling were studied. Osteoblasts and osteoclasts are derived from precursors originating in the bone marrow (19). Interaction among cells mediated by the EphB4 receptor on osteoblasts and the EphrinB2 ligand on osteoclasts generates bidirectional anti-osteoclastogenic and pro-osteoblastogenic signaling into respective cells, potentially facilitating the transition from bone resorption to bone formation (20). This local regulation may contribute to the control of osteoblast differentiation and bone formation at remodeling, and possibly also modeling, sites. In the present study, in order to mimic the *in vivo* environment and the process of bone remodeling, osteoblasts from the jawbones of newborn mice and osteoclasts induced from RAW 264.7 macrophage cells were successfully co-cultured. The effects of Pg-LPS on these cells, and the potential use of Pg-LPS, were then studied.

## Materials and methods

### Animals and chemicals

Female and male newborn Kunming mice (<48 h old) were obtained from the Jilin University Animal Center (Changchun, China). No metabolic or systemic diseases were observed in the mice. Pg-LPS was purified in our laboratory from *Escherichia coli* O55:B5 (Sigma, St. Louis, MO, USA). This study was approved by the ethics committee of Jinlin University (Changchun, China).

### Isolation and culture of osteoblasts

Osteoblasts were isolated sterilely from small specimens of mouse jawbone. Bone fragments (~1 mm^3^) were washed three times with Phosphate buffer saline (PBS) and digested in 0.25% trypsin-EDTA for 10 min. The enzymatic reaction was stopped by adding an equal volume of Dulbecco’s modified Eagle’s medium (DMEM; Gibco, Carlsbad, CA, USA) with 10% fetal bovine serum (FBS; Gibco). Washing of fragments was repeated three more times. The fragments were then placed in the cell culture dish and cultured in DMEM supplemented with 10% FBS and 1% penicillin/streptomycin in a humidified atmosphere containing 5% CO_2_ at 37ºC. When cells covered ~80% of the cell culture dish, conventional digestion and passage were conducted. The medium was changed every two days after being passaged and the cells were ready to use until they were passaged to the third generation. The morphology of the osteoblasts was observed under an inverted phase contrast microscope (Axiovert 200; Zeiss, Göttingen, Germany).

### Osteoblast identification

The isolated osteoblasts were identified through alkaline phosphatase (ALP) staining and the observation of calcium nodes. Elevated ALP expression is one of the most widely used markers for mature osteoblasts. ALP staining was performed using the Burstone method. Prior to observation, the original culture medium was removed and the attached cells were fixed with 10% (v/v) formalin/PBS for 10 min at 4ºC and stained using the substrate naphthol AS-BI phosphate coupled with Fast Blue RR diazonium salt at 37ºC. To perform the observation of calcium nodes, the third generation of osteoblasts, which was cultured for three weeks, was also examined under an inverted phase contrast microscope.

### Induction and culture of osteoclasts

Osteoclasts were induced from RAW 264.7 cells, which were purchased from the China Center for Type Culture Collection (CCTCC, Wuhan, China). During the induction period, RAW 264.7 cells were seeded in a 6-well culture plate at a density of 1×10^4^ cells/well and left overnight. The cells were subsequently treated with 50 ng/ml RANKL to induce osteoclasts, and the culture medium of DMEM supplemented with 10% FBS and 1% penicillin/streptomycin was replaced every two days. The osteoclasts were induced successfully after being cultured for six days.

### Osteoblast-osteoclast co-culture system

The isolated third generation osteoblasts were seeded in the previously mentioned well of induced osteoclasts at a density of 2×10^5^ cells/well. The co-cultured osteoblasts-osteoclasts were treated with 75 ng/ml Pg-LPS for 24 h. Cells cultured without the addition of Pg-LPS were used as the control. The morphology of the co-cultured cells was observed under an inverted phase contrast microscope.

### Protein expression of EphB4 and EphrinB2

EphB4 and EphrinB2 protein expression in the induced osteoclasts and Pg-LPS-treated and untreated co-cultured osteoblasts-osteoclasts were determined by western blot analysis and immunofluorescence staining using antibodies directed at the respective proteins. For western blot analysis, cells were harvested and lysed and the total protein content was determined using a BCA protein assay kit (Beyotime, Beijing, China). The lysate with 30 mg protein was loaded onto SDS-polyacrylamide gel for electrophoresis and transferred to a nitrocellulose membrane. The membranes were blocked in 5% nonfat dried milk for 45 min at 37ºC and then incubated overnight with 1:1000 mice anti-EphB4 monoclonal antibody (Santa Cruz Biotechnology, Inc., Santa Cruz, CA, USA), and 1:1000 mice anti-EphrinB2, monoclonal antibody (Santa Cruz Biotechnology, Inc.) at 4ºC. The membranes were washed three times in TBST and incubated with the corresponding secondary anti-mouse antibody (Santa Cruz Biotechnology, Inc.) conjugated with horseradish peroxidase (HRP) at room temperature for 45 min. The detected protein signals were measured using an enhanced chemiluminescence (ECL) kit (Beyotime).

### Gene expression of EphB4 and EphrinB2

To further evaluate the expression of EphB4 and EphrinB2, changes in gene expression of EphB4 and EphrinB2 were examined by quantitative reverse transcription-polymerase chain reaction (qPCR). Sequences of the primers for target genes are shown in Table I. According to the manufacturer’s instructions, total RNA was extracted from samples using TRIzol reagent (Invitrogen, Carlsbad, CA, USA) and converted into complementary DNA (cDNA) using a ReverTra Ace^®^ qPCR RT kit (Toyobo, Osaka, Japan). A CFX96™ real-time PCR detection system (Bio-Rad, Hercules, CA, USA) was used to perform the quantitative real-time PCR reaction. The ΔΔCt-value method was used to calculate the relative expression values and all samples were analyzed in triplicate.

### Statistical analysis

Data are expressed as the mean ± standard deviation (SD). An unpaired Student’s t-test was used to test the significance of the observed differences between the study groups. A value of P<0.05 was considered to indicate a statistically significant difference.

## Results

### Identification of osteoblasts

**Figure 1:**
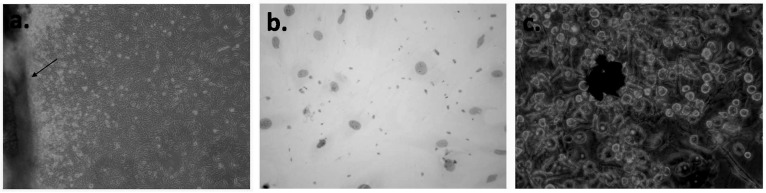
(a) Morphology of the isolated osteoblasts. The arrow shows the sclerite of jawbone; (b) ALP staining with numerous visible particles showing a clear positive effect; (c) high-density black nodular aggregates with varying size visible in the observation of calcium nodes. ALP staining and the observation of calcium nodes confirmed the successful isolation of osteoblasts. ALP, alkaline phosphatase.

The morphology of the osteoblasts is shown in Fig. 1a. After being cultured for five days, cells around the mouse jawbone fragments increased significantly. They became concentrated and certain tissue fragments began to fuse. After seven days, the morphology was varied and the majority of cells were triangular or polygon-like. With increased time, the numbers of osteoblasts increased and the cells were purified through repeated washing and digestion (Fig. 1a).

The ALP staining showed a clear positive effect (Fig. 1b). Many reddish-brown particles were visible in the cells. A large number of high-density black nodular aggregates of varying size were seen during the observation of calcium nodes (Fig. 1c).

### EphrinB2 expression of the induced osteoclasts

**Figure 2:**
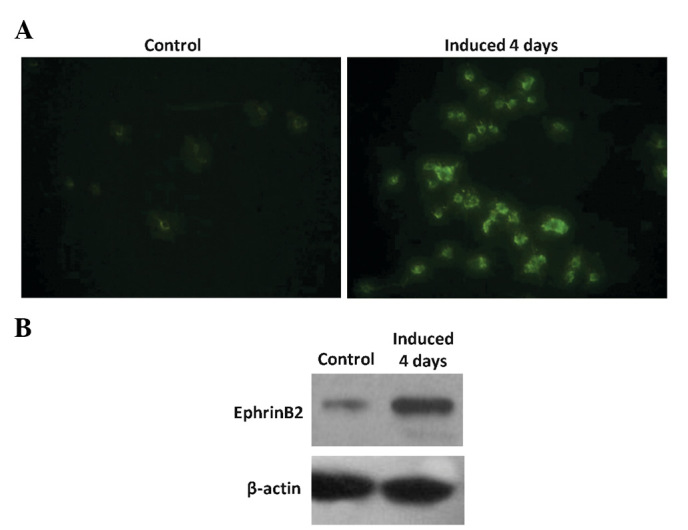
Comparison of EphrinB2 expression between the RAW 264.7 cells and the induced osteoclasts after four days, analyzed by (A) immunofluorescence staining and (B) western blot analysis. The images clearly show an increased expression of EphrinB2 in the osteoclasts and confirm the successful induction of osteoclasts.

The immunofluorescence staining (Fig. 2a) and western blot analysis (Fig. 2b) clearly show that the expression of EphrinB2 was higher in the induced osteoclasts than in the control cells.

### Morphological observation of the co-cultured osteoblast-osteoclast system

**Figure 3:**
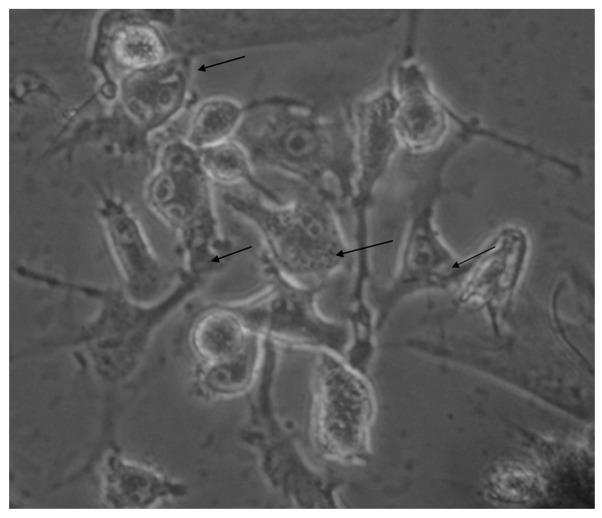
Images of the co-cultured osteoblasts-osteoclasts. The black arrow shows multinucleated osteoclasts and the black arrow shows the triangular osteoblasts.

Direct contact between osteoblasts and osteoclasts was used in the present study. The results indicate that the isolated osteoblasts and induced osteoclasts grew well when co-cultured (Fig. 3).

### Protein expression of EphB4 and EphrinB2

**Figure 4:**
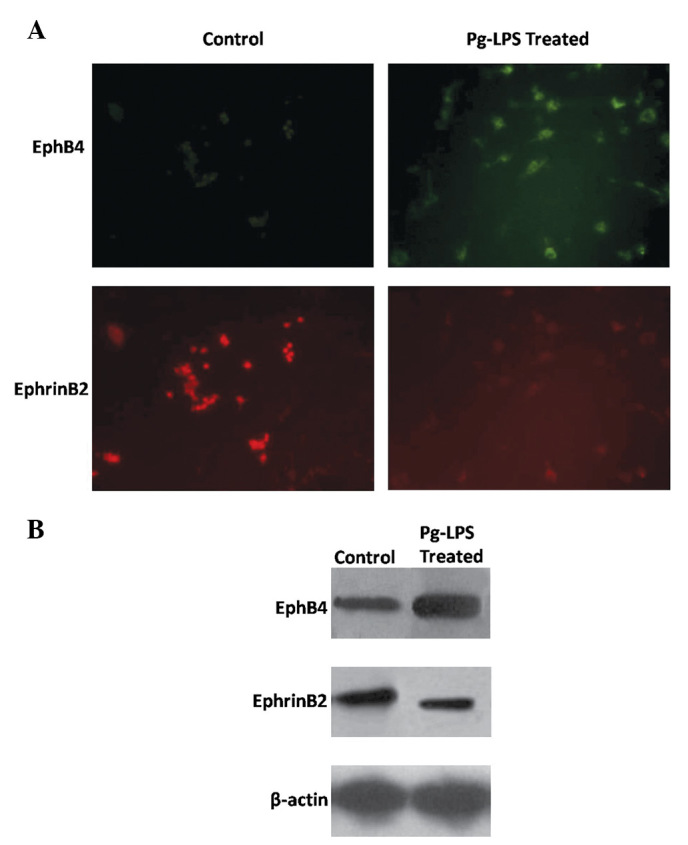
Images of (A) immunofluorescence staining and (B) western blot analysis. After being treated with Pg-LPS at a concentration of 75 ng/ml for 24 h, the expression of EphB4 in the osteoblast-osteoclast co-culture increased while that of EphrinB2 decreased. Pg-LPS, *Porphyromonas gingivalis* lipopolysaccharide.

As shown in Fig. 4, immunofluorescence staining and western blot analysis were conducted to study the changes in the expression levels of EphB4 and EphrinB2 proteins in the osteoblast-osteoblast co-culture. After being treated with Pg-LPS at a concentration of 75 ng/ml for 24 h, the expression of EphB4 increased, while that of EphrinB2 decreased.

### Gene expression of EphB4 and EphrinB2

**Figure 5:**
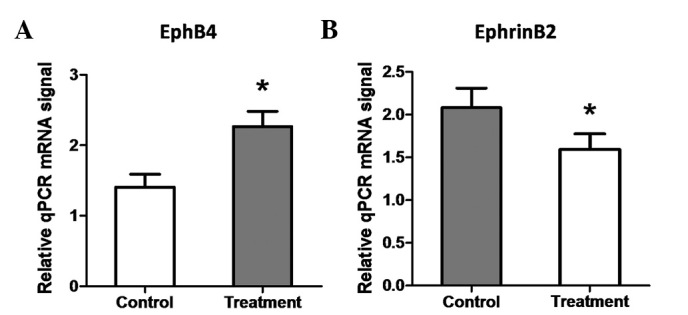
Gene expression of (A) EphB4 and (B) EphrinB2 in the osteoblast-osteoclast co-culture. These results were in line with those for the protein expression. When mediated with Pg-LPS, gene expression of EphB4 was significantly promoted while that of EphrinB2 decreased. Pg-LPS, *Porphyromonas gingivalis* lipopolysaccharide.

The gene expression of EphB4 and EphrinB2 was detected. The results show that the relative EphB4 mRNA expression level was significantly increased in the Pg-LPS-treated osteoclast-osteoblast co-culture compared with that in the control (Fig. 5a; P<0.05). However, EphrinB2 mRNA expression was significantly decreased in the Pg-LPS-treated co-culture compared with that in the control (Fig. 5b; P<0.05). Therefore, the gene studies are in line with those on protein expression.

## Discussion

In the present study, the effects of Pg-LPS on osteoblast-osteoclast bidirectional EphB4-EphrinB2 signaling were investigated. The results show that Pg-LPS increased the expression of EphB4 while inhibiting the expression of EphrinB2.

Our results show that many reddish-brown particles following ALP staining were visible in the cells. A large number of high-density black nodular aggregates of varying size were seen in the observation of calcium nodes (Fig. 1c). Triangular or polygon-like cells centered on the scattered aggregates, thus leading to the formation of calcium nodes. ALP staining and the observation of calcium nodes confirmed the successful isolation of osteoblasts.

RAW 264.7 cells, from Abelson murine leukemia virus-induced tumors, are osteoclast precursor cells derived from mice and are considered to represent the early differentiation stages of the osteoclast precursor (21). Expression of EphrinB2 is one of the indicators of induced mature osteoblasts. Hence, to verify the successful induction of osteoclasts, two complementary assays (immunofluorescence staining and western blot analysis) were employed to monitor the changes in EphrinB2 (Fig. 2). The results showed that the expression of EphrinB2 was significantly increased compared with that in the control group. Thus, the osteoclasts were successfully induced.

Direct contact between osteoblasts and osteoclasts was employed in the present study, in order that certain receptors which exert their impact through direct cell membrane contact were able to function. The co-cultured osteoblast-osteoclast system made it possible to mimic the real environment *in vivo*. After being treated with Pg-LPS at a concentration of 75 ng/ml for 24 h, the expression level of EphB4 increased, while that of EphrinB2 decreased. This result showed clear effects of Pg-LPS on osteoblast-osteoclast bidirectional EphB4-EphrinB2 signaling. Osteoblasts and osteoclasts are derived from precursors originating in the bone marrow (19). The interaction among cells mediated by the EphB4 receptor on osteoblasts and the EphrinB2 ligand on osteoclasts generates bidirectional anti-osteoclastogenic and pro-osteoblastogenic signaling in respective cells, potentially facilitating the transition from bone resorption to bone formation. The present study is consistent with a report by Kubo *et al*(20).

When mediated with Pg-LPS, the gene expression of EphB4 was significantly promoted while that of EphrinB2 was inhibited. EphrinB2, involved in reverse signaling into osteoclast precursors, is associated with the differentiation of osteoclasts. Forward signaling through EphB4 into osteoblasts promotes osteogenic differentiation. Contact between EphrinB2 and EphB4 inhibited the formation of osteoclasts, thus promoting the formation of osteoblasts. The results of the present study indicate that Pg-LPS regulates bidirectional EphB4-EphrinB2 signaling. Therefore, the differentiation of osteoblasts was promoted, while the differentiation of osteoclasts was inhibited. This regulation is considered to be an effective therapeutic approach for the treatment of bone-related diseases. Hence, this study may contribute to the control of osteoblast differentiation and bone formation at remodeling, and possibly also modeling, sites.

In conclusion, when treated with Pg-LPS, the EphB4 receptor on osteoblasts and the EphrinB2 ligand on osteoclasts may generate bidirectional anti-osteoclastogenic and pro-osteoblastogenic signaling into respective cells and potentially facilitate the transition from bone resorption to bone formation.

## Figures and Tables

**Table I:** Sequences of the primers used in the qPCR analysis.

Gene	Sequences
β-actin	F: GGACTTCGAGCAGGAGATGG R: GCACCGTGTTGGCGTAGAGG
Ephb4	F: CCCCAGGGAAGAAGGAGAGCTG R: GCCCACGAGCTGGATGACTGTG
EphrinB2	F: ACTCCAAATTTCTACCTGGACAAG R: GAACCTGGATTTGGTTTTACAAAG

qPCR, quantitative reverse transcription-polymerase chain reaction; F, forward; R, reverse.
